# Magnetic Field Influence on the Microwave Characteristics of Composite Samples Based on Polycrystalline Y-Type Hexaferrite

**DOI:** 10.3390/polym14194114

**Published:** 2022-09-30

**Authors:** Svetoslav Kolev, Borislava Georgieva, Tatyana Koutzarova, Kiril Krezhov, Chavdar Ghelev, Daniela Kovacheva, Benedicte Vertruyen, Raphael Closset, Lan Maria Tran, Michal Babij, Andrzej J. Zaleski

**Affiliations:** 1 Institute of Electronics, Bulgarian Academy of Sciences, 72 Tsarigradsko Chaussee, 1784 Sofia, Bulgaria; 2 Department of Physics, Faculty of Mathematics and Natural Science, Neofit Rilski South-Western University, 66 Ivan Mihailov Str., 2700 Blagoevgrad, Bulgaria; 3 Institute of General and Inorganic Chemistry, Bulgarian Academy of Sciences, Acad. Georgi Bonchev Str., bld. 11, 1113 Sofia, Bulgaria; 4 Greenmat, Chemistry Department, University of Liege, 11 Allée du 6 Août, 4000 Liège, Belgium; 5 Institute of Low Temperature and Structure Research, Polish Academy of Sciences, Ul. Okólna 2, 50-422 Wroclaw, Poland

**Keywords:** Y-type hexaferrite, magnetic properties, microwave properties, reflection losses, external magnetic field

## Abstract

Here, we report results on the magnetic and microwave properties of polycrystalline Y-type hexaferrite synthesized by sol-gel auto-combustion and acting as a filler in a composite microwave-absorbing material. The reflection losses in the 1–20 GHz range of the Y-type hexaferrite powder dispersed homogeneously in a polymer matrix of silicon rubber were investigated in the absence and in the presence of a magnetic field. A permanent magnet was used with a strength of 1.4 T, with the magnetic force lines oriented perpendicularly to the direction of the electromagnetic wave propagation. In the case of using an external magnetic field, an extraordinary result was observed. The microwave reflection losses reached a maximum value of 35.4 dB at 5.6 GHz in the Ku-band without a magnetic field and a maximum value of 21.4 dB at 8.2 GHz with the external magnetic field applied. The sensitivity of the microwave properties of the composite material to the external magnetic field was manifested by the decrease of the reflected wave attenuation. At a fixed thickness, *t*_m_, of the composite, the attenuation peak frequency can be adjusted to a certain value either by changing the filling density or by applying an external magnetic field.

## 1. Introduction

The continuing development of microwave (MW) technologies and the extensive use of high-frequency electromagnetic radiation, not only in various industries but also in medicine and everyday life, such as designing “smart” cities, could lead to a massive increase in electromagnetic interference that could damage sensitive electronic devices and affect human health in ways that are yet to be fully understood. During the past decade, the necessity for the large-scale use of the gigahertz range has resulted in the development of novel functional materials with qualitatively novel properties. Serious efforts have been directed towards producing materials that attenuate (absorb and screen) microwave radiation with high efficiency in order to alleviate problems related to electromagnetic interference and to control its biological impact [1,2,3]. With the continuous innovations in information technologies, higher expectations have been put forward for portable, implantable and wearable electronic devices. The new trends in developing electromagnetic (EM) devices are focused on miniaturization, integration and multifunctionality. However, all of this has led to increased EM radiation pollution, which interferes with the regular operation of precision electronic components; these problems are becoming very serious [4,5].

On the other hand, EM radiation may also heat human cells or interfere with the intrinsic EM field of the human body; thus, harming human health [6,7,8].

These are the main reasons driving the development of various advanced materials for EM wave absorption. The parameters indicating the quality of EM wave absorbers are high reflection losses (RL), small thickness, wide bandwidth and low density [9,10,11,12,13].

Recently, the attention of researchers in this field has been attracted by a large variety of nanomaterials (e.g., magnetic materials and magnetic composites), mainly due to the possibility of controlling their EM wave absorption properties by varying the shape and size of nanoparticles or the structure and composition of nanocomposites [14,15,16,17,18,19].

In view of responding to the growing requirements of the materials intended for such applications, researchers have increasingly focused their attention on developing magnetic composites based on M-, Y-, Z- and U-hexaferrites [20,21]. Hexagonal ferrites are ferrimagnetic materials that are widely investigated for microwave absorption in the GHz frequency band. These materials exhibit strong magnetocrystalline anisotropy, very good high-frequency magnetic properties, large resistivity, good chemical stability and low cost [22,23,24]. In general, a pure hexagonal ferrite exhibits low microwave absorption efficiency and a rather narrow absorbing bandwidth. This is due to their relatively weak EM attenuation ability and a large inequality in magnetic and dielectric losses. To solve these problems and to prepare hexagonal ferrite materials with very good absorbing properties, efforts have been directed towards reducing the materials’ dimension and particle size [25,26], doping with rare earth or transition metal ions [27,28] and combining hexagonal ferrites with other absorbing materials to form composites [29].

Thus, it has been clearly demonstrated that some multiferroic materials, such as the Y-type hexagonal ferrites, could have practical applications with very good reflection losses and absorption bandwidth [30].

This is why the last decade saw strong research efforts geared towards studying the structural and magnetic properties of, and particularly, the magnetic phase transitions in Y-type hexaferrites. This is explained by the magnetoelectric effect at room temperature observed in some of them and the consequent potential practical applications of these compounds as multiferroics.

In this paper, the effects of substituting magnetic Ni^2+^ cations with non-magnetic Zn^2+^ cations in Ba_0.5_Sr_1.5_Zn_2−x_Ni_x_Fe_12_O_22_-based composites on their room temperature microwave behavior, together with the influence of applying an external magnetic field, were studied. The results are presented and discussed concerning the following two Y-type hexaferrite compositions: Ba_0.5_Sr_1.5_Zn_0.5_Ni_1.5_Fe_12_O_22_, Ba_0.5_Sr_1.5_ZnNiFe_12_O_22_, Ba_0.5_Sr_1.5_Zn_1.2_Ni_0.8_Fe_12_O_22_ and Ba_0.5_Sr_1.5_Zn_2_Fe_12_O_22_.

## 2. Materials and Methods

Powder samples of Ba_0.5_Sr_1.5_Zn_0.5_Ni_1.5_Fe_12_O_22_, Ba_0.5_Sr_1.5_ZnNiFe_12_O_22_, Ba_0.5_Sr_1.5_Zn_1.2_Ni_0.8_Fe_12_O_22_ and Ba_0.5_Sr_1.5_Zn_2_Fe_12_O_22_ were prepared by a modified citric acid sol-gel auto-combustion technique using stoichiometric amounts of the precursors. The synthesis procedure is described in detail in [31], and further information on the phase content analysis is given in [32]. In brief, the corresponding metal nitrates were used as starting materials; a citric acid solution was slowly added to the mixed nitrates as a chelator to form stable complexes with the metal cations. The solution was then slowly evaporated to form a gel; thus, it turned into a fluffy mass and burned in a self-propagating combustion manner. The obtained auto-combusted powders were homogenized and thermally treated at 800 °C for three hours. Finally, the Y-type hexaferrite powders were produced as follows: The powders were pressed into bulk pellets with a diameter of 16 mm. The pellets were annealed at 1170 °C in air for 10 h. After cooling down to room temperature, the pellets were ground to obtain the powder materials for the magnetic measurement. For the microwave measurements, the powders were homogenized and used as a filler for composite preparations.

The composite samples were prepared by dispersing the magnetic Y-type hexaferrite (Ba_0.5_Sr_1.5_Zn_0.5_Ni_1.5_Fe_12_O_22_, Ba_0.5_Sr_1.5_ZnNiFe_12_O_22_, Ba_0.5_Sr_1.5_Zn_1.2_Ni_0.8_Fe_12_O_22_ and Ba_0.5_Sr_1.5_Zn_2_Fe_12_O_22_) powders (fillers) in commercial silicon rubber (Mastersil, ASP, Sofia, Bulgaria) as a polymer matrix. Two types of composite samples were prepared as follows: **A** is a series of samples with amounts of magnetic filler of 1.5 g per 1 cm^3^ of silicon rubber (Ba_0.5_Sr_1.5_Zn_0.5_Ni_1.5_Fe_12_O_22_, P1A; Ba_0.5_Sr_1.5_ZnNiFe_12_O_22_, P2A; Ba_0.5_Sr_1.5_Zn_1.2_Ni_0.8_Fe_12_O_22_, P3A; Ba_0.5_Sr_1.5_Zn_2_Fe_12_O_22_, P4A) and **B** is a series of samples with amounts of magnetic filler of 1.8 g per 1 cm^3^ of silicon rubber (Ba_0.5_Sr_1.5_Zn_0.5_Ni_1.5_Fe_12_O_22_, P1B; Ba_0.5_Sr_1.5_ZnNiFe_12_O_22_, P2B; Ba_0.5_Sr_1.5_Zn_1.2_Ni_0.8_Fe_12_O_22_, P3B; Ba_0.5_Sr_1.5_Zn_2_Fe_12_O_22_, P4B). The samples were molded into a toroidal shape with an outside diameter of 7 mm, an inner diameter of 3 mm and a thickness of 4 mm. A referent sample of identical dimensions (denoted as R) made of silicon rubber was only used to study the polymer matrix behavior in the respective microwave range.

The microwave (MW) measurements were conducted using a Hewlett-Packard 8756A microwave scalar network analyzer in a frequency range of 1–20 GHz. To determine the MW characteristics of the composites, a technique was employed whereby the electromagnetic wave (TEM) impinges normally on a single-layer absorber backed by a perfect conductor [33]. The toroidal samples were tightly fitted into a 50-Ω coaxial measurement cell (APC 7) backed by a perfect conductor (short-circuit measurement). During part of the measurements, an external magnetic field was applied using a permanent magnet, providing a flux density of 1.4 T. The magnetic force lines were perpendicular to the direction of the electromagnetic wave propagation. The magnetic flux density in the air gap in the coaxial line was 0.3 T, as measured by a Model 475 Gaussmeter with an HMNT-4E04-VR Hall sensor. The hysteresis measurements were conducted at 300 K by a physical property measurement system (PPMS, Quantum Design Inc., San Diego, CA, USA). Scanning electron microscopy (SEM TESCAN LYRA I, with an EDX detector, Bruker Quantax, TESCAN, Brno, Czech Republic) was used to determine the samples’ morphology and the particles’ distribution in the silicon rubber.

## 3. Results and Discussion

**Figure 1:**
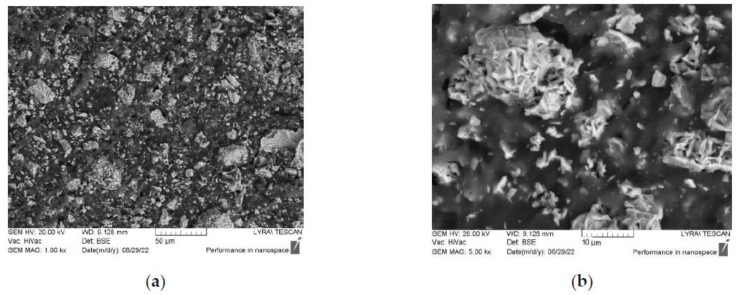
SEM images of (**a**) sample P1A and (**b**) zoomed area of a cluster.

**Figure 2:**
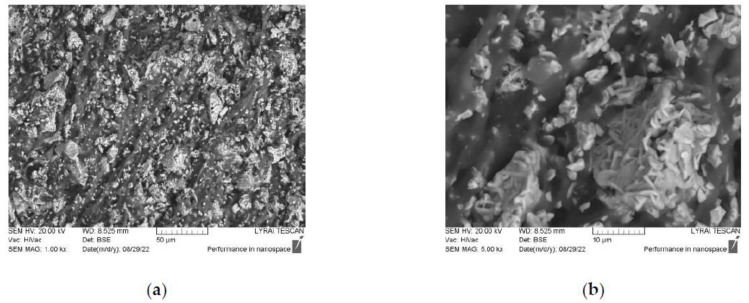
SEM images of (**a**) sample P4B and (**b**) zoomed area of a cluster.

Figure 1 and Figure 2 illustrate the microstructure of the cut P1A and P4B samples. They have different filler densities and different ferrite compositions, but they appear very similar. The SEM images show that the particles are of a hexagonal shape, typical for hexaferrites, and are well agglomerated to form clusters of different sizes and shapes. These clusters are randomly distributed in the volume of the toroidal samples. Our previous investigations have shown that the average particle size is around 600 nm, with the size being within the 200–1000-nm range [34]. Since the same synthesis technique was used for all of the samples, it was only natural to assume that all of them would have a broad particle size distribution. Therefore, they are in a mixed mono- and poly-domain state, since the critical size for a mono-domain state for these types of materials is around 500 nm. Thus, it seemed speculative to discuss the size effect in this case where the size distribution was so broad. This was the main reason why our attention was focused on the Zn:Ni substitution in the samples and its influence on the magnetic and microwave properties rather than on the particle size effect.

**Figure 3:**
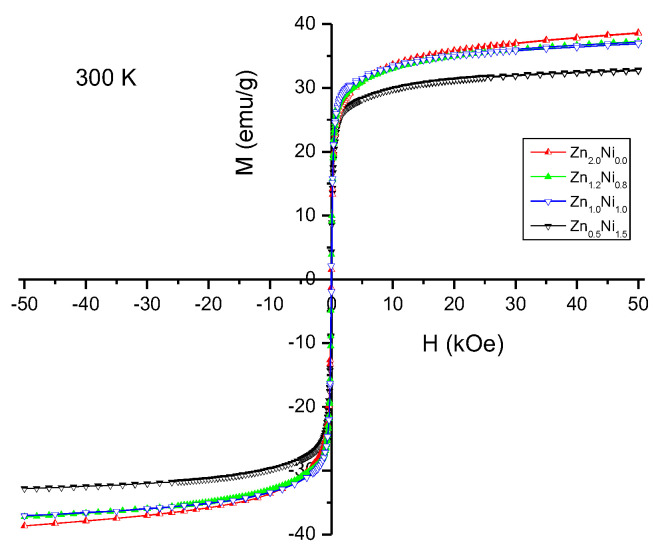
Hysteresis curves of Ba_0.5_Sr_1.5_Zn_2−x_Ni_x_Fe_12_O_22_ (*x* = 0, 0.8, 1.2, 1.5) at room temperature.

Figure 3 presents the hysteresis curves of the Ba_0.5_Sr_1.5_Zn_0.5_Ni_1.5_Fe_12_O_22_, Ba_0.5_Sr_1.5_ZnNiFe_12_O_22_, Ba_0.5_Sr_1.5_Zn_1.2_Ni_0.8_Fe_12_O_22_ and Ba_0.5_Sr_1.5_Zn_2_Fe_12_O_22_ powders. The curves are very narrow, with a coercivity of about a few oersteds. The magnetization values at a magnetic field of 50 kOe (here referred to as saturation magnetization) as a function of the Zn:Ni cation ratio of the samples are presented in Table 1.

It is noteworthy that the sample with the highest content of nickel ions, which bear magnetic moments, has the lowest value of magnetization (50 kOe). This should be related to the known preference of the non-magnetic Zn^2+^ cations to occupy tetrahedral positions, whereas the magnetic Ni^2+^ cations prefer octahedral positions [35]. This entails the migration of iron cations between crystallographic sites with octahedral and tetrahedral oxygen configurations and is accompanied by a corresponding change in the magnetic structure. We also found a similar behavior at low temperatures in our earlier study [31]. One can also see that as the content of the non-magnetic Zn^2+^ cation is increased, the process of saturation is shifted to the higher values of the external magnetic field.

**Figure 4:**
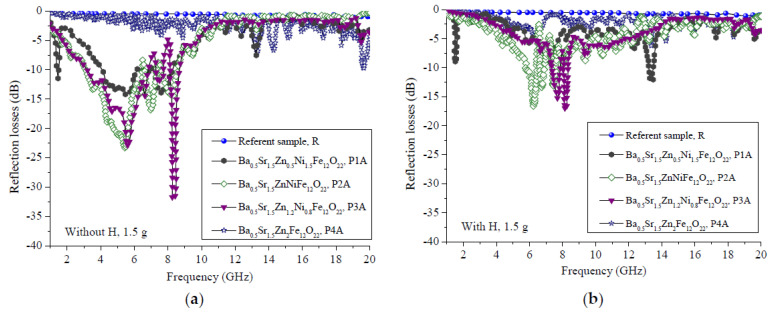
Reflection losses of the **A** series of samples with a filler ratio of 1.5 g per 1 cm^3^ (**a**) with a magnetic field and (**b**) without an external magnetic field applied.

**Figure 5:**
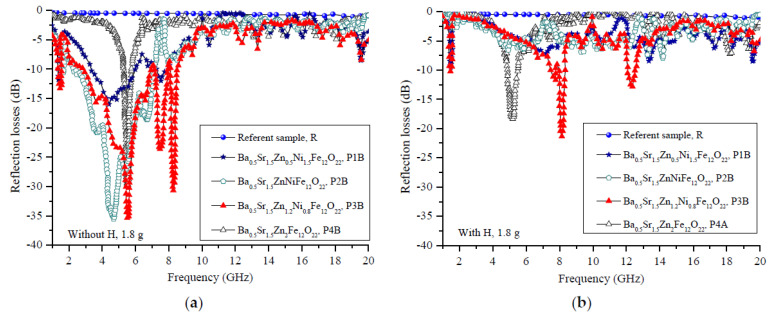
Reflection losses of the **B** series of samples with a filler ratio of 1.8 g per 1 cm^3^ (**a**) with a magnetic field and (**b**) without an external magnetic field applied.

Our investigations were mainly aimed at measuring the microwave characteristics of the composite samples, in which the prepared powder material was dispersed in a polymer matrix (silicon rubber). Figure 4 presents the changes in the reflection losses (*R_L_*) in the sweeping frequency range of 20 GHz for the composite **A** series samples with amounts of magnetic filler of 1.5 g per 1 cm^3^ of silicon rubber (P1A, P2A, P3A and P4A) and the control polymer sample of the same thickness (4 mm) without a filler (R). Figure 5 presents the changes in the reflection losses (*R_L_*) in the sweeping frequency range of 20 GHz for the composite **B** series samples with amounts of magnetic filler of 1.8 g per 1 cm^3^ of silicon rubber (P1B, P2B, P3B and P4B) and the control polymer sample of the same thickness (4 mm) without a filler (R). Our previous experience has shown that the silicon rubber is transparent to electromagnetic waves in this microwave region [36,37], as it is confirmed in Figure 2 for the control (R). Consequently, in the case of a hexaferrite/silicon rubber composite, the microwave properties are due to the hexaferrite only. The curves representing *R_L_* were obtained under the conditions of an electromagnetic wave incident perpendicularly to the surface of toroidal samples backed by a perfect conductor. According to the transmission line theory for such a case, the value of *R_L_* as a function of the normalized input impedance is given by [38,39,40] and is as follows:

(1) $$R_{L}\left(dB\right)=20log\left|\frac{Z_{in}-Z_{0}}{Z_{in}+Z_{0}}\right|,$$

where

(2) $$Z_{in}=Z_{0}\sqrt{\frac{\mu_{r}}{\varepsilon_{r}}}tanh\left[j\frac{2\pi }{c}\sqrt{\mu_{r}\varepsilon_{r}}fd\right]$$

where Z*_in_* is the input impedance of the absorber; Z_0_ is the impedance of free space; *c* is the speed of light in free space; *f* is the electromagnetic wave frequency; *d* is the absorber thickness; $\mu_{r}$ and $\varepsilon_{r}$ are the complex permeability and permittivity, respectively.

An *R_L_* value of −10 dB corresponds to a 90% attenuation of the electromagnetic wave. In general, materials with *R_L_* values below −10 dB could be considered suitable microwave absorbers for practical applications [41].

At certain thicknesses and frequencies, minima are observed in the reflected wave. This takes place when the thickness of the absorber layer satisfies the quarter-wave thickness criterion, described by the quarter-wave theory [42,43] as follows:

(3) $$t_{m}=\frac{nc}{4f_{m}\sqrt{\left|\varepsilon_{r}\mu_{r}\right|}}\left(n=1,3,5,\ldots \right)$$

(4) $$f_{m}=\frac{nc}{4t_{m}\sqrt{\left|\varepsilon_{r}\mu_{r}\right|}}\left(n=1,3,5,\ldots \right)$$

where *t_m_* and *f_m_* are the matching thickness and the peak frequency [44].

To assess the EM wave attenuation, the intrinsic properties of the samples have to be accounted for. The incident microwave energy can generate heat in the material during the interaction of the electromagnetic field with the molecular and electronic structure of the material; the incident microwave energy can generate heat in the material. In this way, this process can convert the incident EM waves into thermal energy, and, finally, the energy will be dissipated.

In a sample, multiple scattering on inhomogeneities can take place [45]. Thus, by varying the material structure, in our case, the density of the hexaferrite particles, one could extend the EM wave propagation path within the sample and improve the absorption capacity of the EM wave absorber [46]. One can conclude that changing the electromagnetic parameters can also enhance the intrinsic EM absorption capabilities.

In the case reported, we measured the MW characteristics of two types of composite samples—with different powder compositions and with different weight ratios. We also added an external magnetic field perpendicular to the propagation of the electromagnetic wave to observe changes in the wave attenuation.

For both the **A** and **B** series of samples, two types of measurements were made—with and without an external magnetic field. The magnetic force lines of this field were perpendicular to the direction of the electromagnetic wave propagation; one would expect that the wave attenuation would be higher due to the orientation of the spins’ magnetic moments. The results from the microwave measurements are opposite. In both cases, for the **A** and **B** series, the reflection losses decreased as a consequence of applying an external magnetic field. For the **A** series (Figure 4) without a magnetic field, the minimal reflection was observed at 8.2 GHz with an *R_L_* = −31.7 dB. In the case with a magnetic field, the best value for the reflection losses was observed at 8.1 GHz with an *R*_L_ = −17 dB. An analogous behavior was identified for the **B** series (Figure 5), but the value of the reflection losses was higher and shifted to the lower frequencies due to the higher hexaferrite content in the composite samples. The minimal reflection without a magnetic field was observed at 4.6 GHz with an *R_L_* = −35.5 dB, and it was observed with a magnetic field at 8.1 GHz with an *R_L_* = −21.4 dB.

**Figure 6:**
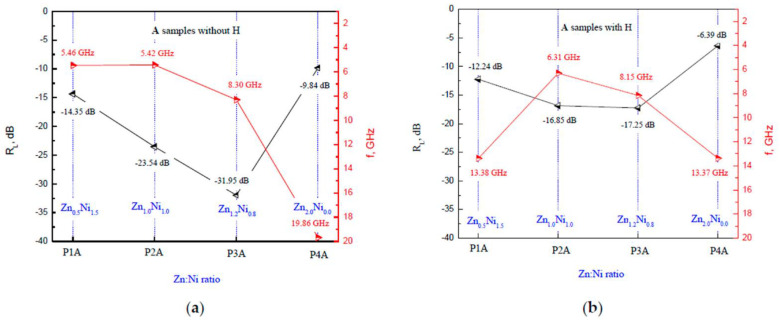
The main reflection loss peaks for the **A** series of samples depending on the Zn:Ni ratio (**a**) without a magnetic field and (**b**) with an external magnetic field applied.

Following the main conclusion of the influence of the magnetic field on the reflection losses, the influence of the Zn:Ni ratio on them was discussed in Figure 6. For the **A** series samples, the main peak with the best value was observed for the Zn:Ni ratio of 1.2:0.8 (Zn_1.2_Ni_0.8_) at 8.3 GHz with an *R*_L_ = −31.9 dB without an external magnetic field. With the magnetic field applied, the sample with the best attenuation was of the same ratio of Zn_1.2_Ni_0.8_ at 8.1 GHz with an *R*_L_ = −17.2 dB.

**Figure 7:**
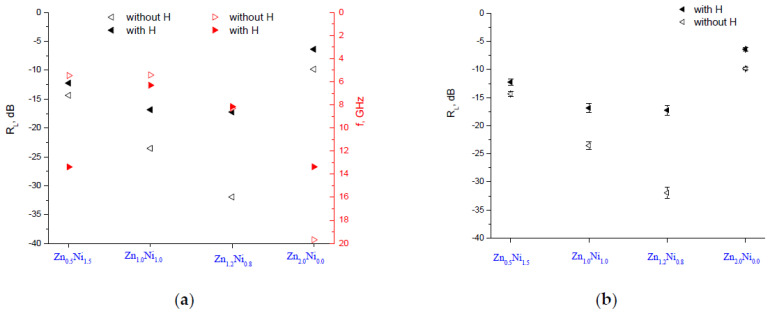
The main reflection loss peaks for the **A** series of samples depending on the Zn:Ni ratio. (**a**) Comparative view with and without a magnetic field and (**b**) an *R_L_* with a standard deviation.

A comparative view of the peaks for the **A** series of samples depending on the Zn:Ni ratio with and without a magnetic field is shown in Figure 7a. It is clear that applying the external magnetic field deteriorates the absorbing characteristics; namely, the attenuation decreases. Figure 7b shows the averaged results of ten measurements, with the standard deviation being 5%.

**Figure 8:**
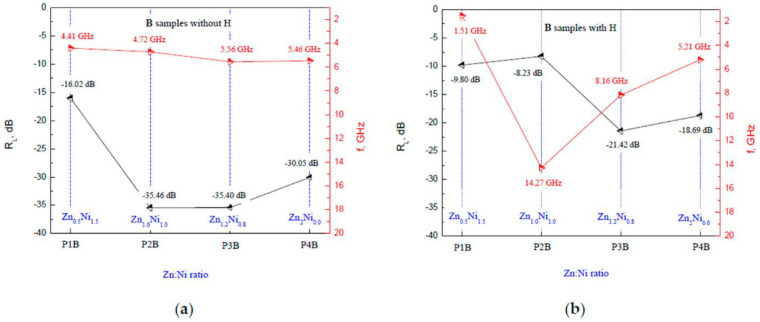
The main reflection loss peaks for the **B** series of samples depending on the Zn:Ni ratio (**a**) without a magnetic field and (**b**) with an external magnetic field applied.

For the **B** series of samples, shown in Figure 8, two main peaks were observed with the same value of reflection losses as follows: for the ratio of Zn:Ni=1:1 at 4.7 GHz and for the ratio of Zn_1.2_Ni_0.8_ at 5.6 GHz with an *R_L_* = −35.4 dB without an external magnetic field. When the magnetic field was applied, the sample with the best attenuation was that with the same ratio of Zn_1.2_Ni_0.8_ at 8.2 GHz with an *R_L_* = −21.4 dB. Compared to the **A** series, the attenuation characteristics are improved, but, again, the external magnetic field plays a negative role and decreases the attenuation.

**Figure 9:**
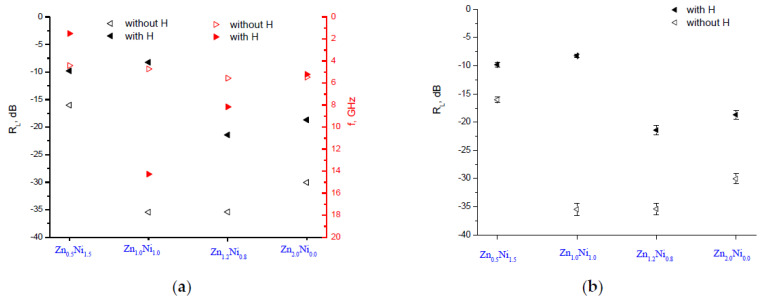
The main reflection loss peaks for the **A** series of samples depending on the Zn:Ni ratio (**a**) without a magnetic field and (**b**) with an external magnetic field applied.

A comparative view of the peaks is shown in Figure 9a for the **B** series of samples depending on the Zn:Ni ratio with and without a magnetic field. A similar behavior was observed when the external magnetic field was applied; the absorbing characteristics deteriorated. Figure 9b shows the results of ten repeated measurements. Here, again, the average value is obtained with a standard deviation of 5%. The most likely explanation for this error is the measurement setup since the samples were fixed mechanically without glue, so that minor differences might have arisen in their positioning.

One can, therefore, conclude that the material’s saturation magnetization was raised by decreasing the degree of substitution with Ni, which, in turn, raised its reflection losses. Additionally, an optimal Zn:Ni ratio, namely, i.e., Zn_1.2_Ni_0.8_, existed there.

These results are opposite to the results observed by us for a Z-type hexaferrite (Sr_3_Co_2_Fe_24_O_41_) under the same experimental conditions [20]; applying a magnetic field resulted in an increased attenuation. In the reported case, for all of the investigated samples, the values of the reflection losses decreased when a magnetic field was applied. Thus, one might speculate that the external magnetic field changes the samples’ permeability to negative or positive [47], depending on whether the material’s resonance frequency is above or below the frequency range of the measurement. Additionally, for all of the samples, a magnetic phase transition from a helicoidal to a ferrimagnetic spin order occurs around room temperature, which was demonstrated in our earlier study [31,34]. This could play an important role in determining the microwave characteristics under an external magnetic field. Accordingly, applying a magnetic field most probably makes the sample more transparent, depending on the intrinsic properties of the Y-type hexaferrite.

## 4. Conclusions

Y-type hexaferrite materials were studied with different Ni substitutions, namely, Ba_0.5_Sr_1.5_Zn_2_Fe_12_O_22_, Ba_0.5_Sr_1.5_Zn_1.2_Ni_0.8_Fe_12_O_22_, Ba_0.5_Sr_1.5_ZnNiFe_12_O_22_ and Ba_0.5_Sr_1.5_Zn_0.5_Ni_1.5_Fe_12_O_22_. The magnetic measurements at room temperature showed that the samples with an increased Ni substitution exhibited a decreased saturation magnetization. This effect impacted the results of the measurements of the composite samples’ microwave characteristics when a Y-type hexaferrite was used as a filler material dispersed in a polymer matrix. It was estimated that composite samples with a higher hexaferrite content had better microwave properties, with the optimal Zn:Ni ratio for the best results being 1.2:0.8. The highest value of the reflection losses, *R_L_* = −35.4 dB, was measured at 5.6 GHz without applying an external magnetic field. When a perpendicular external magnetic field was applied, the absorbing properties of both types of samples changed dramatically; namely, all main peaks were shifted to higher frequencies and the reflection losses decreased. The maximum value of 21.4 dB was found at 8.2 GHz with the external magnetic field applied. Thus, one might assume that the magnetic field makes the composite samples more transparent to the electromagnetic waves in this specific frequency range. This behavior should be related to the intrinsic properties of Y-type hexaferrite materials.

## Figures and Tables

**Table 1:** Magnetization values at a magnetic field of 50 kOe for the samples depending on the Zn:Ni cation ratio.

300 K	Zn_0.5_Ni_1.5_	ZnNi	Zn_1.2_Ni_0.8_	Zn_2_Ni_0_
saturation magnetization, emu/g	32.82	37.07	37.25	38.56

## Data Availability

Not applicable.
